# Scenes, symbols and social roles: raising the curtain on OSCE performances

**DOI:** 10.1007/s40037-020-00593-1

**Published:** 2020-06-05

**Authors:** Gerard J Gormley, Jennifer L Johnston, Kathy M Cullen, Mairead Corrigan

**Affiliations:** Centre for Medical Education, Queen’s University Belfast, Belfast, UK

**Keywords:** OSCEs, assessment, performance

## Abstract

**Introduction:**

Objective structured clinical examinations (OSCEs) are a complex form of assessment, where candidates can interact with ‘patients’ in a constructed socio-clinical encounter. Conceptualizing OSCEs as a complex socially and culturally situated activity offers important research affordances. There are concerns that OSCEs may encourage more strategic ‘tick-box’ candidate behaviours and have a potential negative impact on learner identity formation. This study explored, at a micro-level, the social roles and behaviours occurring within the OSCE triad of simulated patients, candidates and examiners. We used a theoretical framework drawn from Goffman’s dramaturgy metaphor.

**Methods:**

OSCE candidates, examiners and simulated patients were invited, consented and recruited using maximal variation sampling. Participants were allocated to a summative OSCE circuit that had unobtrusive video cameras. Video footage of 18 stations was transcribed. Analysis was interpretative and iterative until a rich and thick description was achieved.

**Results:**

Focusing on elements of Goffman’s dramaturgy metaphor, we foregrounded our analysis by considering the *performers, costumes, props* and the *theatre* of the OSCE. A combination of symbols, both physical and semiotic, was used to construct and maintain layered roles and identities within this tightly defined socio-clinical setting. Informed by this foregrounding, we then considered the social interactions and behaviours within the OSCE: *‘Creating the right impression?’, ‘A performance of contradictions?’ *and *‘Simulated patients: patients or props?’*

**Discussion:**

In the pursuit of standardization, OSCEs have potential to mediate less desirable test-taking behaviours that are not entirely patient-centric, and beyond this may have an impact on professional identity. Whilst OSCE checklists provide objectivity, they have potential to promote a presentation of *self* that is in tension with good medical practice. The certainty of checklists needs to be looked at afresh in order to better reflect the many uncertainties that doctors face in real clinical practice. This research opens up new ways of thinking and enhancing future assessment practices.

## Introduction

Objective structured clinical examinations (OSCEs) are a widely used assessment tool in medical education. They are a constructed phenomenon, aiming to simulate aspects of clinical practice, allowing candidates to demonstrate their clinical skills and behaviours [1, 2]. To date, a psychometric discourse has largely dominated OSCE-related research, with reliability and precision considered as highly desirable characteristics [3–5]. While such positivist research has conferred many insights into this form of assessment, it falls short of capturing the complex sociocultural processes underpinning OSCEs [6]. There is a need to illuminate the highly contextual and socially embedded aspects of this behavioural assessment tool [7].

Typically, OSCE stations are socially situated activities where candidates interact with ‘*patients’ *[1, 2], portrayed by simulated patients [8]. Their highly structured format and reliance on checklists have been criticized for potentially encouraging strategic ‘tick-box’ test-taking behaviours among undergraduate medical students, failing to prepare them for real-life variable and complex patient encounters [5, 9, 10]. We challenge the truism that assessment *drives* learning (i.e. acting more as an external mediator on learning), instead offering a pedagogical viewpoint that assessment *is* learning in action. From this pedagogical position, we require a deeper understanding of the sociocultural interactions within OSCEs. Influenced by not only psychometric but also pedagogic discourse, a more complex understanding would help inform future development of OSCEs and their role in medical students’ professional socialization.

The aim of our research was to explore the social roles, interactions and behaviours occurring within the OSCE triad of simulated patient, candidate and examiner in a summative OSCE. This context, rather than a formative examination, was critical to our research aim.

## Theoretical orientation

In 2003, Hodges [7] described OSCEs using Erving Goffman’s dramaturgical metaphor [11], casting students in the role of doctors, clinicians as examiners and simulated patients as patients. Hodges identified the need for empirical research using Goffman’s conceptual framework, positing that OSCEs are an important site of socialization and therefore clinical identity [7]. In *The Presentation of Self in Everyday Life*, Goffman reasoned that during social interactions, individuals utilized means in an attempt to exert control over the perceptions of others about their identity [11]. Goffman used theatre as an interesting metaphor to explain his theory. In his dramaturgical metaphor he described individuals as actors in a play who put on a show for others. He drew similarities between stage acting and the performance of professional roles.

Goffman’s depiction of interaction as drama and ritual is derived from the sociological theory of symbolic interactionism [12]. This theory focuses on the symbolism of objects and language for creating meaning between individuals based on their interpretation of the symbols. It analyses how individuals ‘negotiate’ social situations to produce new meanings and to manage the impression that others have of them. Goffman distinguishes between the *self *which is how we perceive ourselves (internal) and our *identity* which is how others perceive us (external) [11]. He regards the *self* as multifaceted, and capable of performing and producing different aspects of oneself depending on the situation or encounter. The theories of symbolic interactionism and dramaturgy lend themselves to a micro-analysis of OSCEs and their artefacts, for example examiner checklists, and how they produce certain behaviours between individuals. We therefore chose Goffman’s dramaturgical metaphor as an analytical lens in this study [11].

## Methods

### Ethical and governance approval

This study received ethical approval by the Research Ethics Committee (School of Medical, Dentistry and Biomedical Sciences, QUB; A14/06). A pilot was conducted to explore the feasibility of the study. Prior written informed consent was obtained from all participants in this study.

### Study setting and OSCE context

The study was carried out in a UK medical school, which follows a 5-year undergraduate curricular model. Clinical-based teaching occurs throughout the curriculum, but with a greater emphasis in years 3–5. An end-of-year 3 summative OSCE was used for the purposes of this study, as it marked an important transition in students’ clinical teaching and assessment. Candidates participate in an 18 station OSCE (14 assessment and 4 rest stations) over 2 days with qualified clinicians acting as examiners. Most of the stations have a simulated patient who awards candidates a global score on the humanistic aspects of their performance. As is typical in medical schools in the UK, and many other parts of the world, examiners are usually present with the candidate and simulated patient.

Given that our research focus was on social interactions, we selected OSCE stations that involved dialogue and social interactions between simulated patients and candidates. Three history-taking stations with a simulated patient present were identified:

1. Patient presenting with chest pain
2. Patient presenting with a headache
3. Patient concerned about changes in a pigmented skin mole

Candidates had 6 min for each station: 1 min to read the instructions outside the station and 5 min to perform the clinical task.

### Recruitment and sampling

Third year medical students, lay simulated patients and examiners, who were trained in OSCEs in accordance with the General Medical Council recommendations [13], were invited by e‑mail to participate. Participants’ demographic information (Tab. 1) was used to construct 18 OSCE triads using maximal variation sampling (Tab. 2). Eighteen OSCE triads was chosen to strike a balance between a deep exploration and the broader insights gained by a larger sample. All participants had previously experienced OSCEs.

**Table 1:** Study participant characteristics

Participant	Demographic characteristics
*Candidates (C)*	
C^1^	Male, International, undergraduate entry
C^2^	Male, UK/Irish, graduate entry
C^3^	Male, UK/Irish, undergraduate entry
C^4^	Female, International, undergraduate entry
C^5^	Female, UK/Irish, undergraduate entry
C^6^	Female, UK/Irish, graduate entry
*Examiners (Ex)*	
Ex^1^	Male, Intensive care
Ex^2^	Male, Surgery
Ex^3^	Male, Urology
Ex^4^	Male, General Practice
Ex^5^	Female, General Medicine
Ex^6^	Female, Clinical Biochemistry
*Simulated patients (SP)*	
SP^1^	Male
SP^2^	Female
SP^3^	Female
SP^4^	Male
SP^5^	Female
SP^6^	Female

**Table 2:** OSCE triadic combinations used for purposes of the study

OSCE triad	Candidate, examiner and simulated patient triadic combination
*Chest pain station*	
1	C^1^, Ex^1^, SP^1^
2	C^2^, Ex^1^, SP^1^
3	C^3^, Ex^1^, SP^1^
4	C^4^, Ex^2^, SP^2^
5	C^5^, Ex^2^, SP^2^
6	C^6^, Ex^2^, SP^2^
*Headache station*	
7	C^1^, Ex^3^, SP^3^
8	C^2^, Ex^3^, SP^3^
9	C^3^, Ex^3^, SP^3^
10	C^4^, Ex^4^, SP^4^
11	C^5^, Ex^4^, SP^4^
12	C^6^, Ex^4^, SP^4^
*Suspicious skin mole*	
13	C^1^, Ex^5^, SP^5^
14	C^2^, Ex^5^, SP^5^
15	C^3^, Ex^5^, SP^5^
16	C^4^, Ex^6^, SP^6^
17	C^5^, Ex^6^, SP^6^
18	C^6^, EX^6^, SP^6^

### Data capture

Participants were allocated to a designated exam circuit. At the micro-societal level, Goffman’s dramaturgical metaphor focuses on how individuals engage with and respond to one another in a given social setting [11]. Blumer reasoned that when wanting to capture a deep awareness of the dynamics in a social interaction, first-hand observations are generally more desired [12]. Therefore observation and ethnographies, rather than post-hoc interviews, are typically used to systematically and contextually observe social roles and behaviours. In the setting of a summative OSCE, having a researcher physically present within the close confines of an OSCE station may have undesirable effects on any social interactions. Video capture offers the means of observing social interactions, without the close proximity of a researcher in the social situation under investigation. Therefore data were collected using unobtrusive pre-existing, ceiling-mounted video cameras and microphones.

Participants were not aware which 3 of the 14 assessment OSCE stations were being recorded. Footage captured the ‘1 min reading time’ and the station itself. The 18 triads generated 108 min of video and audio footage, which were transcribed verbatim. Transcripts were compared against footage to ensure accuracy.

### Data analysis

The data analysis was initially iterative with the researchers observing the stations live via a video link and making written notes of their initial observations and thoughts. GJG, JLJ and MC carried out a more in-depth, interpretative and iterative analysis once the video footage was transcribed and cross-checked for accuracy. The units of analysis were the verbal and non-verbal interactions between the simulated patients, examiners and candidates. Video data, transcripts and field notes were analyzed together and coded interpretively. After the first independent viewings and readings, the researchers agreed on dimensions for further focus, drawing upon Goffman’s dramaturgical metaphor [11]. Memo writing took place throughout, with frequent meetings to refine analysis with reference to the conceptual framework. Team reflexivity checks took place regularly to minimize systematic distortion from researcher preconceptions or assumptions. Analysis ended when all researchers agreed a rich, interpretative description had been achieved.

## Results

In addressing our research aim, we initially focused on ‘setting the scene’ and mapping the dramaturgical elements of the OSCE stations. Informed by this foregrounding, we then considered the social interactions and behaviours within the OSCE triads—represented by the following themes: *‘Creating the right impression?’, ‘A performance of contradictions?’ *and *‘Simulated patients: patients or props?’*

### ‘Setting the scene’

**Fig. 1:**
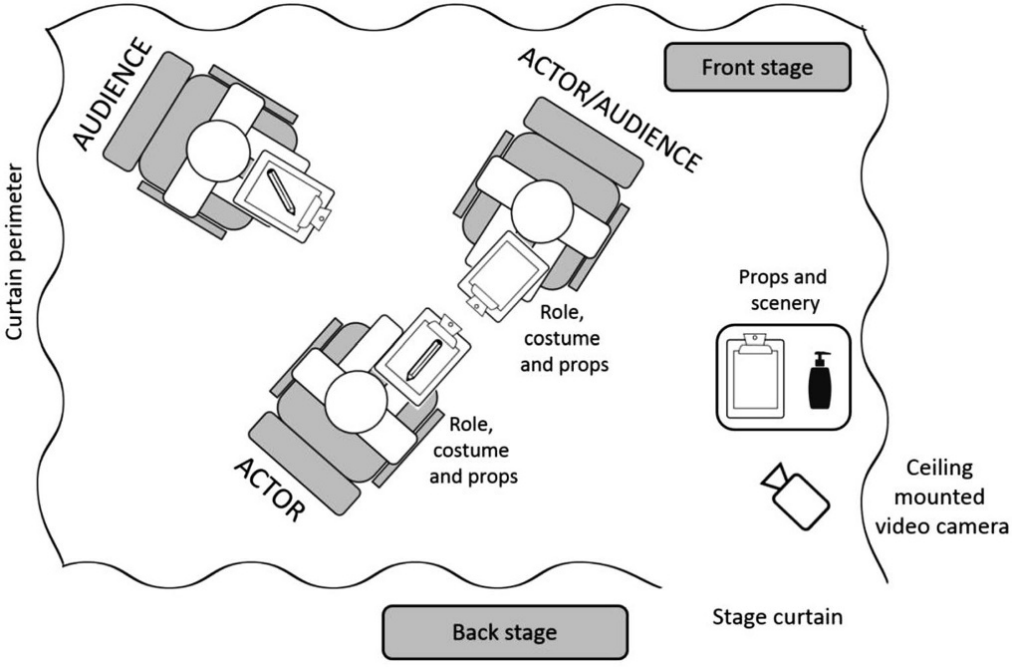
Diagrammatical overview of an OSCE station in relation to Goffman’s dramaturgical framework

A combination of symbols, both physical and semiotic, were used to construct and maintain layered roles and identities within this extraordinary, tightly defined setting. We focused on the *performers, costumes, props *and the *theatre* to map out the various elements of the OSCE stations and how they related to Goffman’s dramaturgical metaphor (Fig. 1).

#### The performers

Candidates became lead *actors* in a performance intended to convince examiners of their competence. Simulated patients were primarily *supporting actors*, giving voice to the tightly scripted character and presence of a surrogate patient. Their role portrayal was mediated by adherence to the SP script provided to them by the OSCE organizers, with few opportunities to deviate from the script. They also had a dual role as *audience* as they were responsible for awarding a global rating score for candidates’ performances. However, as far as the candidates were concerned, the examiners were the *primary audience *given their powerful role in awarding the majority of marks.

#### Costumes and props

Simulated patients were often casually dressed, symbolizing their expected ‘everyman’ role. Candidates wore smart casual clothes, without white coats, mirroring the usual attire of clinicians in the UK. Although not in a clinical area, shirtsleeves were rolled up to their elbows and ties were not worn, reflecting adherence to hospital infection control policy despite the university setting. Stethoscopes hung round the candidates’ neck, even when they were not required, which symbolized the clinicians that students hoped to become and their intention to fit in.

Examiners were dressed smartly for the occasion, and did not comply with infection control policy, nor did they carry stethoscopes. The formality of their dress was in contrast to the informal dress of the simulated patients and the smart casual dress of the candidates. College ties and lanyards branded with their university or hospital were often worn and acted as important symbolic claims to status. These artefacts marked establishment identity, serving to embody authority within the person of the examiner, and to emphasize the solemnity of the occasion.

Several ‘props’ were used to support the performance. The clipboard, which held the examiner’s mark sheet, conveyed authority and power in determining success or failure. The clipboard was often used as a physical barrier to separate the examiner from the simulated consultation. The bell was another ‘prop’ that marked the start and the end of a production that was highly industrialized.

#### The theatre: front and back stage

In any piece of theatre, players prepare themselves *backstage *(area just outside the station where candidates waited before entering the station). Before entering the station, candidates had one minute of reading time in the *‘wings’*. This represented a crucial liminal space in which to gather themselves for the task ahead. While candidates prepared for their entrance, simulated patients and examiners were provided with a break from their repetitive performances. During this ‘interval’, examiners often filled in marking sheets. Occasionally, whispered conversations were held, sometimes about candidates (acting as critical audience members) and other times social trivialities (conducted entirely out of role). Examiners often used their clipboard to avoid conversing, or simulated patients turned their attention to the floor, ceiling or dividing curtain until the bell marked the beginning of the next performance.

Under the gaze of examiners, candidates’ performances were offered up in the confines of the OSCE station (*front stage*). The physical curtains (or room dividers) in the station provided a strong boundary between the actor and audience; between the *front stage* and *backstage*. Passing through the OSCE (stage) curtains signified the transition from putting on and taking off the candidate’s character; the transition from *being* to *performing *and from *self* to* portrayed identity—*transition into their character. Both examiner and simulated patient adopted a more active position, leaning forward as they readied themselves to commence their roles.

### ‘OSCE performances’

#### Creating the right impression?

When candidates entered the station (front stage), impression management particularly came into sharp focus. Pulling back the curtain, candidates introduced themselves while simultaneously applying hand sanitizer. This was always undertaken in a ritualized, at times exaggerated manner with outstretched arms at chest level, in a gesture aimed at ticking the checklist box for ‘hand hygiene’. Initial eye contact was often not with the simulated patient, but rather with the examiner. This tacit acknowledgment of the authority they embodied also had a secondary outcome, placing the simulated patient in a subordinate position. Asking simulated patients for their date-of-birth was a staple of all introductions and was an acknowledgement to patient safety. A well-rehearsed string of introductory questions would often flow from candidates, delivered in a formal fashion. Candidates often attempted to read examiners’ expressions or body language for clues or feedback, gazing at the clipboard which symbolized success or failure in their role.

#### Excerpt from Triad 7

*[Bell sounds; candidates enters station, acknowledges examiner and then turns attention to simulated patient* (*SP). Expresses sanitization foam on hands and washes hands at eye level of SP].*

C^1^: ‘Hello my name is *** and I am a Foundation Level doctor’
SP^3^: *[Sits forward] *‘Good afternoon’
C^1^: ‘Can I confirm your name and date of birth?’ *[Sits on chair]*
SP^3^: ‘My name is Ruth Smith and I am 56’
C^1^: ‘Today I would like to ask you a series of questions to see what brought you in here today. So what brought you here today?’ *[Intermittently looks at examiner]*
Ex^3^: *[Examiner observes checklist and intermittently gazes at candidate]*

#### A performance of contradictions?

OSCEs strive to provide judgements on behaviours that reflect real clinical practice—with competency and compassion being key tenets of what makes a *good doctor*. However, OSCEs have potential to challenge this fundamental assumption. Within the framework of OSCEs, the pressure on candidates’ behaviours to display competency had the potential to suppress more humanistic behaviours. There was a drive to seek information in an efficient manner, when candidates’ *overt speech acts* (i.e. what they said) often were not congruent with their *ungovernable acts* (i.e. the sincerity of how they delivered their overt speech acts) [11]. Here, the simulated patient’s revelation of a family bereavement was met with minimal lip-service and lack of eye contact that lacked empathy.

#### Excerpt from Triad 1

C^1^: ‘Do you have any family history of something like this?’
SP^1^: ‘Yes, well, my father had a heart attack aged 59—he died’
C^1^: *[Looks at note book whilst giving reply] *‘OK; sorry to hear that’ … ‘Do you smoke?’
SP^1^: ‘No’

With the burden on candidates to ‘tick all of the boxes’ within the time-frame of the station, they would often slip into a more *digitized *mode of history taking that lacked compassion.

#### Excerpt from Triad 7

C^1^: *[leaning forward on chair] *‘On a scale of 1–10, with 10 being this worst, how would you score your headache?’
SP^3^: *[Sitting forward in chair; facial expressions of discomfort, holding head] *‘I would say 9 …’
C^1^: *[Rubbing hands] *‘… alright, we will just move on now to talk about your general medical history’

Difficulties in recollecting the next line of questioning led to an awkward pause, or the candidate briefly abandoning role to think aloud. Candidates, who were pressured for time, would adopt strategies of ‘shot-gunning’ rapid-fire questions at the simulated patient sometimes over the concluding bell and replacing proper sentences with a checklist of symptoms that they ticked off on their fingers. All pretence of a *real life* consultation was abandoned in these circumstances.

#### Excerpt from Triad 8

C^2^: *[leaning forward on chair] *‘It’ll be quite fast paced but if you can say yes or no’
SP^3^: *[Leaning forward on chair holding SP instruction sheet] *‘OK. OK’
C^2^: *[Adjusts stethoscope draped around neck] *‘So, you’ve had this headache over the last 3 weeks?’
SP^3^: ‘Yes’ *[Nods head]*
C^2^: …‘any muscle weakness?’
SP^3^: ‘No’ *[Shakes head]*
C^2^: ‘..the water works’?
SP^3^: ‘No’

Analysis of examiners’ behaviours also revealed contradictions. Examiners’ primary role was to observe candidates’ entire performances and make an objective checklist recording. However, the need to accurately complete checklists often attracted a significant proportion of examiners’ *visual *attention towards the checklist and away from candidates’ performances. This meant that examiners appeared to experience more of the aural rather than the visual aspects of the performances.

#### Simulated patients: persons or props?

Simulated patients’ roles within OSCEs are to portray the patients and their experiences, providing a human dimension to the constructed OSCE clinical encounter. Whilst candidates had potential to excel in their patient centeredness, there was a potential drive to tick boxes—which mediated less *person-centric* behaviours in candidates. Such candidate behaviours risked rendering simulated patients more as props rather than treating them as individuals. Working to a predetermined script, simulated patients had relatively little to say, and little agency to engage beyond these bounds. Exchanges between doctors and patients should be constructive, involve active listening and acknowledgment of the patient as a *person *[14].

The treatment of simulated patients as props was encouraged by the structure of some of the OSCE stations, which required the examiner to interrupt the consultation with a question. In response, candidates shifted abruptly out of character and appropriated the common clinical speech genre of case presentation. A speech genre is a particular form of communication which is recognizable to both parties and which accomplishes a particular social purpose—in this case conforming to the traditional medical student/teacher (represented by the examiner) dyad [15]. Candidates used neutral, scientific language in answering questions, and referred to the simulated patient in the third person using linguistic constructions such as ‘*41-year-old male presenting with …*’, rather than the patient’s name. Permission was not sought from simulated patients to speak about them in this third party manner.

#### Excerpt from Triad 12

C^6^: *[Faces examiner, holds chin, scratches back of head, folds arms] *‘… 41-year-old male presented with, first of all, an occipital bad headache …’
SP^4^: *[Looks down at floor, then again at candidate while she summarizes, arms folded]*
Ex^4^: *[looks at notes, looks at candidates and nods] *‘What is the most likely diagnosis, is the last question?’
SP^4^: ‘Raised intracranial pressure’
Ex^4^: ‘That’s fine *[looks at candidates, nods and lowers head] *I have no other questions for you.’

## Discussion

In this highly rigid and constructed form of behavioural assessment, the OSCE triad is a microcosm in which all actors adhere closely to scripts, roles and prescribed activity. Shared semiotic and material symbols facilitate the development of social roles and interactions within this assessment setting. OSCEs thus offer a rich context for the study of learning and identity in medical education.

Each triad role represents a broader constituency: the medical establishment (examiners), patients (simulated patients) and doctors (candidates). Although candidates are under explicit scrutiny, so too are the other groups subject to the bounds and affordances of their socially determined roles. The motivation of all three groups is, in Goffmanian terms, to give and receive affirmation of their roles and identities relative to one another [11]. For candidates in particular, as trainees on the threshold of acceptance into the medical community, triad interactions offer the possibility of affirmation and approval.

### The cast

Simulated patients were constrained by their scripts, portraying in essence a sophisticated prop against which the candidate could demonstrate their skills. Their lack of agency rendered them more subordinate and an uncomplicated conduit for their OSCE script. This is in keeping with the status quo in the UK where it is common practice for simulated patients to occupy a more codified role based largely on standardization of performance. In this educational context, relatively little attention is paid to their agenda, agency or indeed individuality [8, 16–18].

Despite their apparently greater freedom, however, candidates and examiners were also tightly bound by social convention. Candidates have the leading role, engaged in a high-stakes performance with the overriding aim of creating an impression of competence for examiners, their primary audience. This pursuit of competence is in tension with good relational care. While the roles of examiners are similarly circumscribed, they represent primarily the medical establishment, without threat to their role.

### The script

Deviation from the OSCE script is unwelcome; undermining reliability and standardization agendas. Given the value placed on ‘following the script’, semiotic symbols—those related to language—carry enormous importance for the triad. Goffman drew a useful distinction between what someone says (*overt speech acts*) and the message they are actually conveying (*ungovernable acts*). In other words, paralanguage can refute the overt speech act [11]. Candidates’ *ungovernable acts*, directed towards the examiner, transmitted a lack of sincerity in their ‘relationship’ with the SP. Candidates largely focused on their *overt speech act* as this represented their perceived framework of success—ticking checklist boxes within the defined timeframe.

Such predictable patterns of OSCE communication constituted a clear speech genre in tension with real life practice. There, the consultation is dialogic [19]. Patients and doctors co-construct a consultation based on a negotiation of both agents’ expertise and the lived experience of illness [14]. No two illness experiences, or consultations, are the same. Unlike OSCEs, clinical presentations will always have a degree of natural variation. There is a significant contradiction between the speech genre of the OSCE consultation, and any genre of real-life clinical consultation.

### Performing or being?

OSCEs comprise a series of role enactments which are intended to simulate real-life clinical encounters. Yet from the point of view of clinical practice, simulated patient-candidate dyads were led into overtly dysfunctional forms of relating to each other. The industrialized, standardized OSCE often falls short of simulating real practice, offering instead an alternative reality defined by homogeneity and efficiency. Lines become blurred between ‘real’ and ‘constructed’ clinical encounters [20], thus the OSCE begins to take on an agency of its own which can distort identities. The immersive, high-stakes nature of the OSCE means that this constructed reality takes on a significance that goes beyond conscious performance. Students learn to think of themselves as doctors, and to understand the acceptable face of doctoring, by interpreting the validity of their performance through their marks on a checklist. What looks like reality, may become reality, with students learning to interact with patients in real life as they do in this most valued form of assessment. It is important to appreciate the nuance that in performing in OSCEs, triad members are not simply *role playing* but by performing a specific identity also are *doing* and *being*.

Clinical practice is complex and dynamic. OSCEs, with their tightly defined scripts and consequent role-play, can fall short of recreating the complex emotional, social and psychological landscapes of patients’ illnesses, or to consider how candidates should best navigate these with patients [14]. The natural to and fro of real life consultations is suppressed [21]. Such a genre of communication presents a risk of diminishing the importance of empathy, humanism, partnership and acceptance of uncertainty. Homogeneity and itemization, hailed in psychometric terms, come potentially at a professional cost. Standardization is a constraining force, which must be balanced against its gains and losses [22, 23].

### Strengths and limitations

This area has been under-investigated to date, and it is a strength that the data were generated from *summative* OSCEs. We acknowledge, however, some limitations. The generality of OSCEs means that results should transfer well to other contexts. Local differences in the running of the exam may, however, limit transferability (e.g. in context where the examiner is not present within the confines of the OSCE station). Furthermore, medical students in this study were mid-way through their undergraduate training. Differences may well be seen with more junior or indeed senior medical students.

Participants may have behaved differently because of the video recording. We feel such a ‘halo’ effect was minimal. Video cameras were inconspicuous and in position *a priori*. Participants were not aware of which triad interactions were recorded. Only on one occasion did an examiner and simulated patient look up to the camera, and this happened at the outset of the overall OSCE. We acknowledge that there is a significant digression in the role of simulated patients in the UK context and other parts of the world. This provides an opening for further enquiry.

We have deliberately not used the term ethnography to describe the methodology, since this implies in-depth observation over a longer period of time than was possible here. Finally, we chose to focus on the triad in this study. There are many others involved in the set-up and ‘backstage’ running of OSCEs which we will explore in a further study. Similarly, we chose stations involving simulated patients and a significant ‘talking’ component in the form of history taking. There are other types of OSCE stations which may offer different affordances.

### Practice points

Co-working in partnership with patients is a cornerstone of modern medical practice [24]. Employing simulated patients in OSCEs focuses clinical care on the patient [25]. By working in partnership with simulated patients, and with real patients, in designing and delivering OSCEs could focus greater attention on candidate/patient rather than candidate/examiner social interactions. Replacing tightly bounded scripts with social and clinical information about the patient that require simulated patients to improvise, but which remain anchored in assessment objectives, may provide more flexible and adaptable social interactions. The highly itemized examiner checklist could be replaced by a domain or ‘semi global’ scoring based approach that would promote more realistic doctor-patient interactions. ‘Semi global’ scores (i.e. aggregating several checklist items into one broad domain or semi global score) can have a positive effect on the quality metrics in an OSCE [3]. Incorporating the social dynamics of OSCEs into station development and the training of examiners and simulated patients is also an important next step.

Finally, OSCEs represent just one method from a wide range of assessment tools that are used in facilitating judgements on clinical competency. In addition to more knowledge-based assessment tools (e.g. multiple choice questions) and work-based assessment tools (e.g. miniCEX), OSCEs contribute to a multimethod approach in assessing clinical competency. Work-based assessment tools afford judgements on students competencies, in a more naturalistic and contextual setting—as opposed to the more constructed nature of OSCEs as highlighted by our research. Therefore it is important to consider, from an assessment blueprint perspective, that learning outcomes are best aligned to the most suitable assessment tool. Not all clinical scenarios are best suited to be assessed within an OSCE context, and indeed may be more appropriately assessed by work-based assessment methods. Furthermore, they may be fewer opportunities of certain clinical events in the workplace (e.g. breaking bad news or managing an acutely unwell patient) to facilitate work-based assessment with large cohorts of medical students. Therefore, such high acuity clinical events would be more appropriately assessed within an OSCE framework.

## Conclusions

This study presents an exploration of the social roles and interactions, mediated by physical and material symbols, within summative undergraduate medical OSCEs. In the pursuit of standardization and reducing variance, OSCEs have potential to mediate less desirable test taking behaviours that are not entirely patient-centric. Whilst OSCE checklists provide objectivity to assessment, they also encourage a presentation of self in tension with that of the ‘*good doctor*’ [25]. The certainty of OSCE checklists needs to be looked at afresh in order to better reflect the many uncertainties and difficulties that healthcare professionals face in clinical practice. Working synergistically with the psychometric research paradigm, such qualitative findings can potentially be transformative in developing the next generation of OSCEs. Whilst OSCEs aim to contribute to professional development, paradoxically they may drive behaviours not in keeping with the spirit of good medical practice. At its most simplistic level assessment *drives* learning but OSCEs may be driving aspects of professional socialization in not such the right direction. If we accept the notion that assessment *is* indeed learning, such insights open up lines of action to enhance future assessment practices and the *learning* that occurs within them.
